# Tuberculous Diseases of Bones and Joints

**Published:** 1912-03

**Authors:** 


**Tuberculous Diseases of Bones and Joints.** By Sir W. Watson Cheyne. Pp. xi, 411. London: Oxford Medical Publications. 1911.

We welcome a second edition of this most valuable book. We have always considered it to be one of the best books—if not the best—on the subject. As the first edition was published in 1895, we are very glad to have it brought up to date by a new edition, which gives the results of a wider experience of the author than the last. We notice the addition of many skiagrams and pictures illustrating the clinical aspects of joint disease, and these are very helpful. In the preface to this new edition Sir Watson Cheyne tells us that operative interference seems to be less often required now than when the first edition of the book was published, *i.e.* nearly seventeen years ago. In the chapter on "Operation *versus* Expectant Treatment," he suggests that more cases now recover without operation because of the better hygienic conditions under which we live at the present time and of the disease becoming less virulent. But he says in his preface that it is especially the frequency of operations at the early period of the disease, and in the absence of suppuration, which has been steadily diminishing of late years. Now, operation in an early stage of the disease and in the absence of suppuration has not been generally regarded as necessary, and it may be that the few surgeons who did practise it have wisely abandoned it. It seems to us probable that many of the cases operated on in this stage would have recovered without operation, and hence that there is no need to suppose that the recovery of similar cases at the present time without operation is due either to improved hygienic conditions or to the disease being of a less virulent form. It is so very difficult in many cases to say how far Nature can be trusted to effect a cure, as we have usually only probabilities to guide us in our decision, and it is quite possible that cases which do well after operation might do as well without. We believe that many cases of tuberculous peritonitis which are said to have been cured by coeliotomy would have recovered equally well without, as it is known that many such cases recover under medical treatment alone.

Sir Watson Cheyne tells us that he has been very disappointed with the results of vaccine treatment, and that he has not seen any greater improvement in joint or bone disease by the use of the vaccine method than he would have expected to follow careful treatment on former expectant lines. But he does not seem to be without some hope of its doing good even after so disappointing an experience, for he continues to employ it in conjunction with operative and other measures. We are not surprised at his disappointment with so unreasonable a method of treatment, and we predict that when the next edition of his book appears he will tell us that he has entirely given up its use.

The author refers very favourably to the treatment of abscess in connection with tuberculous diseases of the joints and spine, by aspirating them, as practised with such success by Drs. Calve and Gauvain at the Cripples' Hospital at Alton. This is not, of course, a new treatment, but it has been brought prominently to the notice of the profession by the publication of a paper on the subject by these two surgeons. Sir Watson Cheyne says he still thinks that when the abscess sac cannot be dissected out the best treatment where the surgeon can be sure of asepsis is to open, scrape and flush them out; but if this cannot be done, he recommends aspiration as practised by Calve and Gauvain.

